# Novel *Saccharomyces cerevisiae*-Loaded Polyvinylpyrrolidone/SiO_2_ Nanofiber for Wound Dressing Prepared Using Electrospinning Method

**DOI:** 10.3390/ma17122903

**Published:** 2024-06-13

**Authors:** Yeon Seo Cho, Hongjun Yoon, Sung Giu Jin

**Affiliations:** Department of Pharmaceutical Engineering, Dankook University, 119 Dandae-ro, Dongnam-gu, Cheonan 31116, Republic of Korea

**Keywords:** *Saccharomyces cerevisiae*, nanofiber, electrospinning, polyvinylpyrrolidone, silicon dioxide

## Abstract

Electrospun nanofibers have been used as wound dressings to protect skin from infection and promote wound healing. In this study, we developed polyvinylpyrrolidone (PVP)/silicon dioxide (SD) composite nanofibers for the delivery of probiotic *Saccharomyces cerevisiae* (SC), which potentially aids in wound healing. PVP/SD composite nanofibers were optimized through electrospinning, and bead-free nanofibers with an average diameter of 624.7 ± 99.6 nm were fabricated. Next, SC, a wound-healing material, was loaded onto the PVP/SD composite nanofibers. SC was encapsulated in nanofibers, and nanofibers were prepared using SC, PVP, SD, water, and ethanol in a ratio of 3:4:0.1:4.8:1.2. The formation of smooth nanofibers with protrusions around SC was confirmed using SEM. Nanofiber dressing properties were physicochemically and mechanically characterized by evaluating SEM, DSC, XRD, and FTIR images, tensile strength, and elongation at break. Additionally, a release test of active substances was performed. The absence of interactions between SC, PVP, and SD was confirmed through physicochemical evaluation, and SEM images showed that the nanofiber dressing contained SC and had a porous structure. It also showed a 100% release of SC within 30 min. Overall, our study showed that SC-loaded PVP/SD composite nanofibers prepared using the electrospinning method are promising wound dressings.

## 1. Introduction

The skin protects internal organs from various external chemical, biological, and mechanical influences. It can be easily damaged by cuts and burns [1,2]. Wound dressings are used to protect wounds from the loss of fluids and harmful exogenous substances [3]. Nanofibers are of great interest in the field of drug delivery because they have a high surface area–volume ratio and allow controlled release of active ingredients [4]. The high surface area–volume ratio and porous nature of these nanofibers make them suitable for applications in vascular transplantation, drug delivery, and wound dressings [5,6]. Nanofibers can be formed by electrospinning technology [7]. Electrospinning technology enables the loading of active substances for heat-sensitive wound healing and is a technology that can manufacture wound dressings more cost-effectively than traditional methods [8]. This technique consists of a syringe pump and a collector containing a dressing solution connected to a high-voltage supply, enabling the fabrication of nanofibrous structures [9]. Wound dressings manufactured by electrospinning are composed of microporous nanofibers whose diameters can be adjusted from nano to micro sizes. This microporous fibrous structure not only provides a large surface area for cells to attach to, but can also increase the loading of active substances [10]. In addition, the microporous structure can quickly release active substances, simultaneously promoting oxygen permeation and preventing body fluid accumulation, making it an ideal structure for wound dressings [11]. Nanostructures with smaller nanofiber diameters provide a higher surface area–volume ratio, and therefore, higher loading capacity and efficiency. As a result, electrospinning technology is a facile method for fabricating nanostructured wound dressings with high surface area–volume ratios [12].

The manufacturing of wound dressings requires a variety of natural and synthetic biopolymers. Electrospinning techniques typically use polymers to provide chain entanglement during the electrospinning process to sustain a polymer jet. Electrospun polymer nanofibers have limited applications because of their limited functionality when prepared using polymers alone. Therefore, research on composites, such as various polymer/inorganic mixtures, is necessary to expand the functionality of nanofibers [13].

Polyvinylpyrrolidone (PVP) is used in various biomedical engineering fields owing to its biocompatibility, excellent mechanical properties, and processability [14]. Many studies have shown that PVP is a suitable polymer for the manufacture of electrospun wound dressings; however, its use is limited in the manufacture of wound dressings when used alone [15,16]. Composites comprising PVP nanofibers with various additives have been reported to provide active material loading and improve mechanical properties. Examples of these additives include reports of using polymer materials such as polyvinyl alcohol, polyurethane, ethyl cellulose, and chitosan, and inorganic materials such as carbon nanotubes, hydroxyapatite, silicone rubber, and mesoporous silica [15,16,17,18]. In this study, we attempted to evaluate the application of silica material.

A variety of bacteria, including gram-positive and gram-negative, have been encapsulated in nanofiber structures. For example, Lactobacillus acidophilus has been added to polyvinyl alcohol and PVP nanofibers to treat bacterial vaginosis and wounds [19,20]. Polyvinyl alcohol and polyethylene oxide nanofibers have also been used for the encapsulation and delivery of intestinal probiotics [21]. However, only a few studies have been conducted on the encapsulation of microorganisms in PVP and inorganic composite nanofibers. *Saccharomyces cerevisiae* (SC) has been reported to have excellent antioxidant and antibacterial effects and has been reported as a new active substance related to wound recovery [22,23]. Therefore, in this study, we attempted to manufacture a wound dressing containing SC that has the function of promoting wound recovery. Electrospinning technology was used to maximize the wound dressing effect by loading heat-sensitive active substances.

Nanocomposite fibers made by adding inorganic substances to polymer nanofibers can add the advantages of inorganic substances, such as enhanced mechanical properties and stability (thermal and chemical), to the polymer properties of flexibility and moisture absorption [24]. In this study, a pharmaceutically applicable silicon dioxide (SD) inorganic material was prepared by directly adding SD particles to a PVP solution easily and conveniently. SC-loaded wound dressings were generated by direct electrospinning of PVP/SD dispersions.

This study aimed to fabricate a nanofiber dressing containing SC with improved mechanical and physical properties and the smallest possible diameter of PVP/SD composite nanofibers while maintaining a uniform nanofiber morphology through electrospinning technology. SD particles were incorporated into PVP nanofibers to achieve better physicochemical properties in terms of wound dressing. The SC-loaded electrospun nanofiber wound dressings were characterized based on their physicochemical, mechanical, and SD release properties. Scanning electron microscopy (SEM), differential scanning calorimetry (DSC), X-ray diffraction (XRD), and Fourier transform infrared spectroscopy (FTIR) were used to determine the physicochemical properties of these SC-loaded PVP/SD nanofibers. In addition, the release characteristics of SC were evaluated.

## 2. Materials and Methods

### 2.1. Materials

*S. cerevisiae* ATCC 18824 (SC) was purchased from Microbiologics (Saint Cloud, MN, USA). PVP and SD were purchased from BASF (Ludwigshafen, Germany) and Evonik (Essen, Germany), respectively. All other chemicals were used as received without further purification.

### 2.2. Preparation of SC

SCs were grown in YPD (1% yeast extract, 2% peptone, 2% glucose) medium at 33 °C and 180 rpm for 48 h. SC was harvested by centrifugation at 2500× *g* for 5 min at 25 °C [25]. Subsequently, the SC was homogenized in an ultrasonic homogenizer and used to manufacture nanofiber dressings.

### 2.3. Preparation of Electrospun Composite SD/PVP Nanofibers

**Figure 1:**
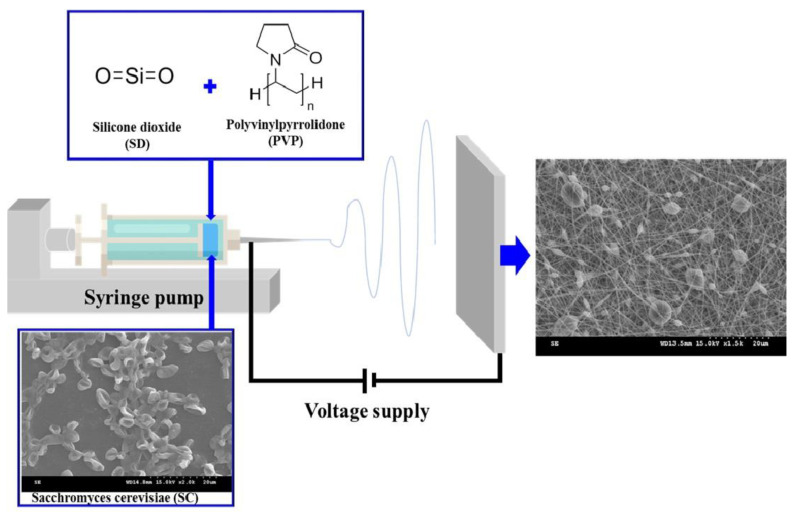
Schematic diagram for manufacturing SC-loaded PVP/SD nanofiber using electrospinning technology.

Each nanofiber wound dressing was manufactured by electrospinning using ESR 100 NanoNC electrospray equipment (Seoul, Republic of Korea). A PVP solution was prepared by completely dissolving PVP in a solvent (an ethanol or ethanol/water mixed solvent) according to the composition in Table 1. In the formulation using SD, SD particles were dispersed in the prepared PVP solution. The dispersion was stirred for 1 h, sonicated for 2 h, and electrospun under stirring to prevent phase decomposition. In addition, when incorporating SC, cultured SC was added to the dispersion prepared previously and dispersed for 1 h. The electrospun dispersion, prepared according to the composition listed in Table 1, was placed in a glass syringe (Hamilton Co., Reno, NV, USA) attached to a digital syringe pump, and a 30-gauge needle was used. The electrospinning process was performed at a nozzle–collector distance of 17.5 cm, injection rate of 0.3 mL/h, and high voltage of 18 kV [26,27]. The fabricated nanofibrous dressings were collected using aluminum foil sheets at room temperature (22–24 °C) and 60% relative humidity [9]. All electrospun dressings were vacuum-dried at room temperature for 24 h. The moisture content of the prepared nanofibers was analyzed using an OHAUS MB27 moisture analyzer (Parsippany, NJ, USA). Samples were placed in the instrument and heated to 110 °C at a constant heating rate (30 s increments of 1 °C) until the samples reached constant mass. The moisture content was controlled to less than 0.5%. A schematic of the electrospun wound dressing is shown in Figure 1.

### 2.4. Physicochemical Properties of Electrospun Composite SD/PVP Nanofibers

Scanning electron microscopy: Images of electrospun nanofibers were obtained using Hitachi S3400 SEM equipment (Tokyo, Japan). Samples coated with platinum under vacuum were observed under an electron microscope at an accelerating voltage of 3 kV [28]. All SEM images of the electrospun nanofibers were analyzed using ImageJ software (version 1.8.0). For each nanofiber, three photographs were analyzed and 100 fiber diameters were manually measured from each photograph. Results were expressed as mean ± standard deviation.

Differential scanning calorimetry: Thermal aspects of the samples were examined using a TA Instruments DSC Q20 (New Castle, DE, USA). Each sample was heated at a rate of 5 °C/min over a temperature range of 50–300 °C [29].

X-ray diffraction: XRD patterns of the samples were recorded by Rigaku XRD Ultima IV (Tokyo, Japan) using filtered Cu Kα radiation at a voltage of 40 kV and a current of 30 mA. Samples were scanned over a 2θ range of 5 to 45°, determined using a step size of 0.02°. The scanning speed was fixed to 1.0 s/step [30].

Fourier transform infrared spectroscopy: FTIR patterns were obtained for samples in the range 4000–450 cm^−1^ using a PerkinElmer Frontier spectrometer (Waltham, MA, USA) [31].

### 2.5. Mechanical Properties of Electrospun Composite SD/PVP Nanofibers

The tensile strength and elongation at break of the nanofiber dressings were measured at room temperature using an AND texture tester MCT2150 (Seoul, Republic of Korea). To evaluate mechanical properties, 2 cm × 3 cm samples of each nanofiber dressing were cut. The tensile strength (maximum weight of the load at dressing rupture) and elongation at break (strain divided by the initial sample length) were calculated [32]. Six specimens were analyzed per nanofiber dressing.

### 2.6. In Vitro Release Study of SC-Loaded Electrospun Composite SD/PVP Nanofibers

SC release from SC-loaded electrospun nanofiber dressings was assessed using the USP Apparatus V paddle-over-disc method [33]. A device consisting of a Hanson Vision Classic 6 paddle and vessel assembly (Los Angeles, CA, USA) and a small disk assembly (APPFIVE-V80, QLA, Telford, PA, USA) was used. The release study was performed in 500 mL of isotonic phosphate buffer (pH 7.4) at 32 ± 0.5 °C at 100 rpm. Samples (1 mL) were collected at 5, 30, 60, and 120 min. SC concentration was determined by measuring the optical density at 600 nm using a GE Healthcare UV-Vis spectrophotometer Ultrospec7000 (Little Chalfont, UK) [34]. SC concentration was determined based on the optical density of the suspension formed by the release of SC from the SC-loaded nanofiber dressing. Moreover, the SC content in SC-loaded nanofibers was equally determined by measuring the optical density at 600 nm. The initial value of cultured SC was set to 100% and the content was confirmed by checking the same optical density value in the SC-loaded nanofiber.

### 2.7. Statistical Analysis

Data were analyzed by ANOVA (SigmaPlot version 11). Differences were considered statistically significant at *p* < 0.05.

## 3. Results and Discussion

### 3.1. Effect of PVP and SD Concentration

In this study, nanofiber dressings loaded with SC as an active material for wound healing and various formulations were manufactured and evaluated using electrospinning. Nanofibers prepared by electrospinning can form porous structures that mimic the natural tissue extracellular matrix and promote epithelial cell adhesion, proliferation, migration, and differentiation [35]. A higher specific surface area can effectively absorb exudate from the trauma surface and stop bleeding while promoting the loading and release of active substances. Additionally, electrospun nanofibers can promote cellular respiration and prevent moisture loss owing to their random arrangement and high porosity (60–90%) [36]. Additionally, they block external microbial invasion and prevent granulation tissue on the trauma surface from growing into the dressing. Because of these advantages, electrospun nanofibers can be used to fabricate desirable wound dressings. Therefore, in this study, we optimized SC to maintain a homogeneous nanofiber shape and produce nanofibers with the smallest possible diameters. Preliminary evaluations established process parameters, such as the applied electrospinning voltage, operating parameters, including the tip-to-collector distance, relative humidity, and feed rate, as well as the concentrations of polymers and additives included in the electrospinning process. PVP, used as a polymer, exhibits excellent biocompatibility, biodegradability, and hydrophilic properties and has been used as a nonionic synthetic polymer for electrospinning [19]. Additionally, PVP is a vinyl polymer with good water and ethanol solubility and highly polar side groups owing to the peptide bond in the lactam ring. SD was used as the inorganic material to manufacture the composite electrospun nanofibers. SD is a pharmaceutically applicable material with a special structure and properties consisting of highly ordered nanosized pores. In addition, SD has excellent chemical stability and biocompatibility; therefore, it is applied not only in the drug delivery field, but also in various biotechnology fields. For example, SD is used in various fields, such as sustained drug release, gene delivery, and biosensing [37].

The goal was to manufacture nanofibers with the smallest diameter while maximizing the amount of inorganic substances in the nanofiber composite and maintaining uniform dispersion of nanoparticles within the composite nanofiber. Nanofibers could be manufactured when the polymer PVP–solvent ratio was adjusted to 4:6 (PVP–solvent weight ratio). SD used as an inorganic material produced nanofibers up to a ratio of 0.1, and no nanofibers were produced at a ratio higher than that. In addition, as an important factor in optimizing the electrospun nanofibers, the solvent composition in the electrospinning solution was changed and evaluated during manufacturing [38].

By electrospinning pure PVP, it was confirmed that the PVP ratio was highest at a high molecular weight–solvent ratio of 4:6 (PVP–solvent weight ratio), forming a nanofiber structure. It has been reported that the incorporation of inorganic materials into nanofibers increases physical strength, thermal stability, and chemical stability [16]. In this study, nanofibers were manufactured by physically mixing SD, an inorganic material that can be used pharmaceutically.

**Figure 2:**
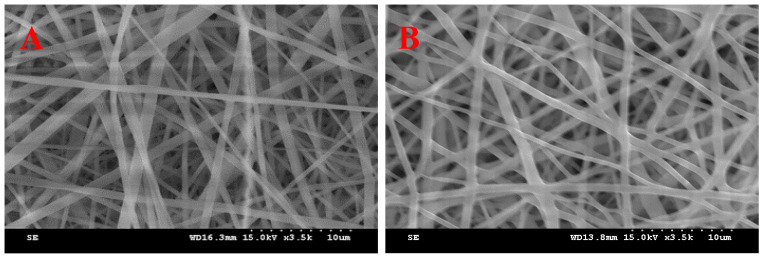

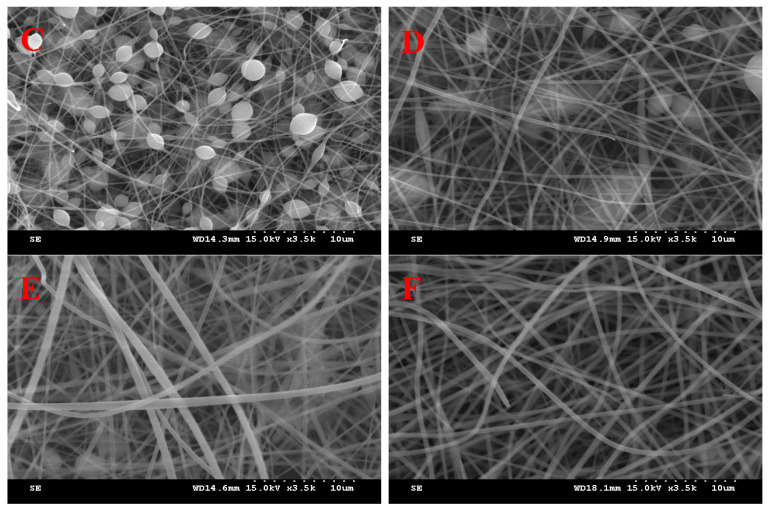
Scanning electron micrograph images of electrospun nanofibers with different formulations: (**A**) I (×3500), (**B**) II (×3500), (**C**) III (×3500), (**D**) IV (×3500), (**E**) V (×3500), and (**F**) VI (×3500).

**Figure 3:**
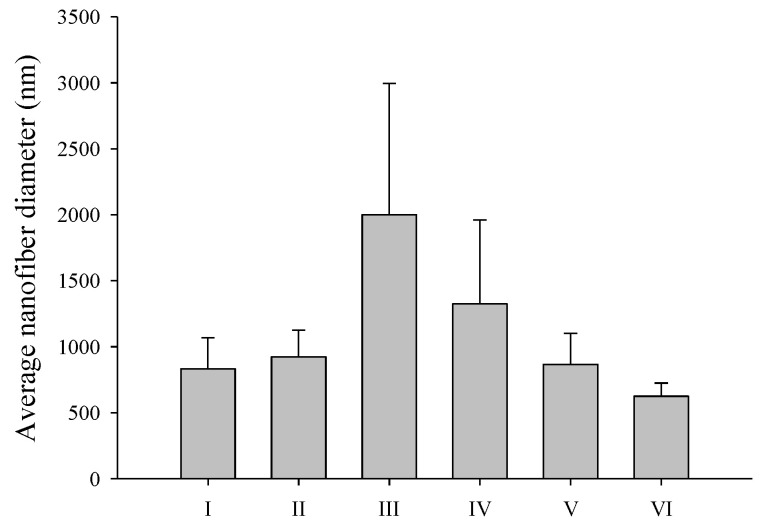
Average diameter of electrospun nanofibers with different formulations.

Formulations I and II in Table 1 were prepared under the same conditions using only 100% ethanol as the solvent, and water was added for SC loading to prepare formulations III to VI with the same composition as formulation II, but with different solvent compositions. Figure 2 shows SEM images of the resulting composite nanofibers. Figure 3 shows the average nanofiber diameter. Nanofiber structures were manufactured in both formulation I without SD and formulation II with SD, and the average nanofiber diameter increased from 832.4 ± 235.4 nm to 924.5 ± 201.1 nm when SD was added, but there was no significant difference. We focused on using only generally regarded as safe (GRAS) solvents to manufacture the nanofiber dressings. Therefore, only water and ethanol were used as solvents. Water and ethanol can be used in the pharmaceutical field and have been used for electrospinning in other studies [39]. Formulation III was evaluated to maintain the same polymer concentration as in formulation II and to evaluate the effect of the type of solvent. Formulations III–VI were prepared by varying the solvent composition and water–ethanol ratio. As shown in Figure 2, nanofibers of formulations III and IV, with high water content, contained bead-like morphological modifications (Figure 2C,D). Beads produced by electrospinning are the result of surface tension that overcomes the forces of jet elongation produced by electrospinning [40]. Previous research suggests that the boiling point of the solvent, vapor pressure, surface tension, and dielectric constant are factors that affect the properties of nanofibers in electrospinning [41]. The bead morphology was eliminated by increasing the proportion of ethanol in the electrospinning solution to promote sustained jet elongation. Therefore, nanofibers in formulations V and VI did not contain beads (Figure 2E and 2F, respectively). Additionally, as the ethanol ratio in the solution increased, the average diameter of the nanofibers decreased, as shown in Figure 3. In the electrospinning method, a high voltage induces a charge in the solution, which is necessary to initiate electrospinning because the surface tension is overcome by the electrostatic forces of the solution. Therefore, the dependence of surface tension on solvent composition is an important variable in electrospinning [40]. By increasing the solvent proportion of ethanol, which has a relatively low surface tension, the ratio of PVP–SD–water–ethanol in formulation VI was 4:0.1:4.8:1.2 (weight ratio), and the average particle size was the smallest, at 624.7 ± 99.6 nm. Therefore, formulation VI, which produced nanofibers with the smallest diameter without bead deformation, was determined to be the optimal composition.

### 3.2. SC-Loaded Electrospun Nanofiber

**Figure 4:**
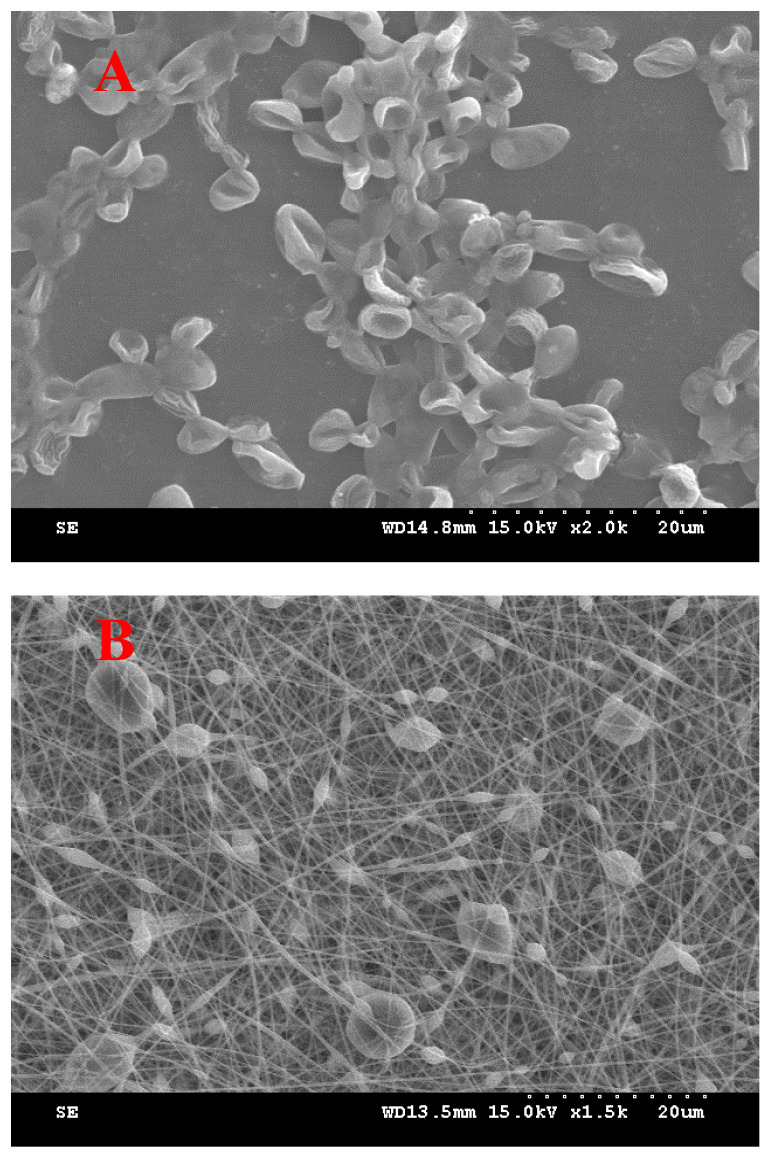
Scanning electron micrograph: (**A**) SC (×2000) and (**B**) SC-loaded PVP/SD nanofiber (formulation VII) (×1500).

To evaluate the SC loading effect, SC, PVP, SD, water, and ethanol in formulation VII were prepared at a ratio of 3:4:0.1:4.8:1.2. The SC ratio was selected through a preliminary evaluation to determine the amount that could be loaded at the highest ratio. When manufactured by adding SC, it was confirmed through SEM that the SC in Figure 4A was loaded onto the nanofiber dressing (Figure 4B), as shown in Figure 4. Compared with the SEM image of SC alone (Figure 4A), it was confirmed that each SC was encapsulated in the middle of the nanofiber, forming a smooth electrospun nanofiber with protrusions around the SC [42].

**Figure 5:**
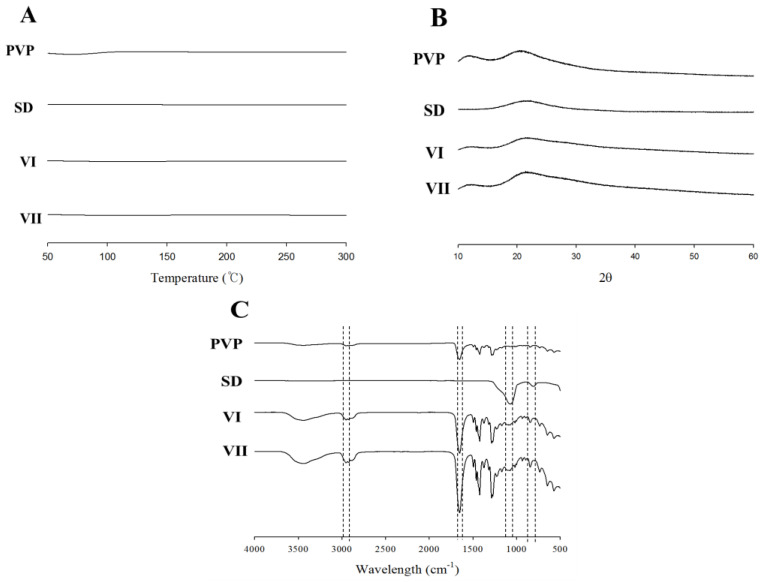
(**A**) DSC graphs, (**B**) XRD patterns, and (**C**) FTIR spectra: PVP, SD, PVP/SD nanofiber without SC (formulation VI), and SC-loaded PVP/SD nanofiber (formulation VII).

DSC, XRD, and FTIR were used for the physicochemical characterization of the electrospun nanofiber dressings containing SC (Figure 5) [43]. To determine the thermal behavior of the SC-loaded nanofiber dressings, endothermic peaks of PVP, SD, SC-free nanofibers (formulation VI), and SC-loaded nanofibers (formulation VII) were compared (Figure 5A). No specific endothermic peaks were observed for PVP, SD, nanofibers without SC, or nanofibers with SC (Figure 5A). These results showed that SC can be encapsulated and exhibit amorphous dislocations in SC-loaded nanofibers. To confirm the crystallinity of the SC-loaded nanofiber dressings, the XRD diffraction peaks of PVP, SD, nanofibers without SC (formulation VI), and nanofibers with SC (formulation VII) were measured (Figure 5B). PVP, SD, nanofibers without SC, and nanofibers with SC did not exhibit any specific crystalline peaks (Figure 5B). This result also showed that, in the SC-loaded nanofibers, SC was encapsulated in the nanofibers and did not crystallize. FTIR spectra of PVP, SD, nanofibers without SC (formulation VI), and nanofibers with SC (formulation VII) are shown in Figure 5C. PVP showed stretching bands at 2944 cm^−1^ (C-H stretching) and 1651 cm^−1^ (C=O stretching). SD exhibited an absorption peak at approximately 1100 cm^−1^ due to the asymmetric stretching vibration of the Si-O-Si bond and a peak at 820 cm^−1^ due to the symmetric strain of the Si-O-Si bond. No particularly different peaks were observed between nanofibers without SC (formulation VI) and those containing SC (formulation VII). Additionally, absorption bands characteristic of PVP and SD were observed (Figure 5C). Therefore, it can be concluded that no strong molecular interactions occur between the constituents in the presence or absence of SC or PVP/SD nanofibers [16,44].

**Figure 6:**
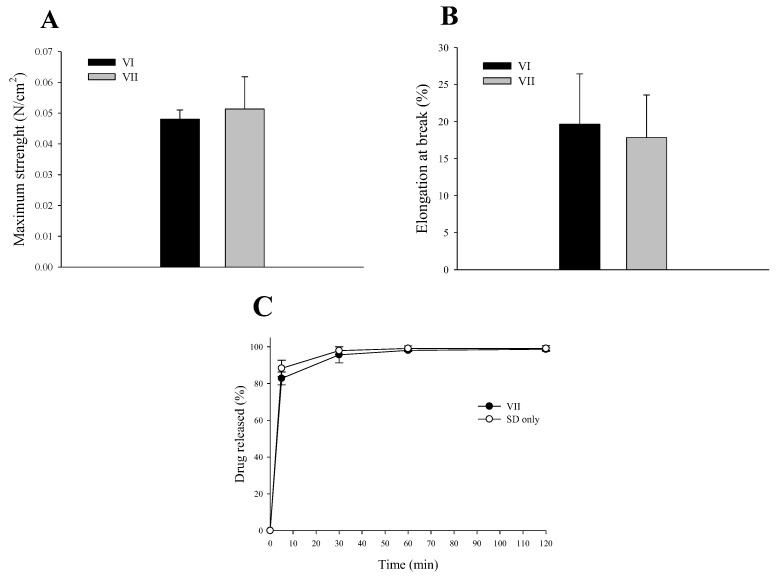
Effect of SC on tensile strength (**A**), break elongation (**B**), and SC released (**C**). Formulation VI was composed of PVP, SD, water, and ethanol. Formulation VII was composed of SD, PVP, SD, water, and ethanol.

The physical strength, tensile strength, and breakage rate of the electrospun nanofiber dressings were evaluated. (Figure 6A,B) The nanofiber dressing containing SC (formulation VII) had an increased tensile strength (Figure 6A) compared to the nanofiber dressing without SC (formulation VI). The elongation at break (Figure 6B) was lower for formulation VII with SC than for formulation VI without SC. However, although significance was verified through statistical analysis, there were no significant differences in tensile strength and elongation at break. Tensile strength and elongation at break were approximately 10 times lower than previous research results on other types of dressings [3]. This suggests that electrospun nanofibers may have weaker physical strength compared to hydrocolloid or hydrogel types. SC release evaluation was performed with a nanofiber dressing of formulation VII prepared with SC, PVP, SD, water, and ethanol in a ratio of 3:4:0.1:4.8:1.2 (Figure 6C). The release of active substances from the PVP/SD nanocomposite nanofibers is an important property for demonstrating the effectiveness of active substances in dressings. When SC was included in the PVP/SD composite nanofiber structure, its release was so rapid that it reached 100% within 30 min. Therefore, the PVP/SD composite nanofiber is a structure that can be loaded with the active material, SC, and exhibits rapid release characteristics, making it a useful structure for application to wound areas.

## 4. Conclusions

This study aimed to demonstrate the feasibility of delivering SC to wound sites by loading them onto PVP/SD composite nanofibers. Composite nanofibers were prepared using PVP/SD, and water and ethanol compositions were optimized to optimize the morphology of the PVP/SD composite nanofibers. The addition of ethanol reduced the surface tension of the electrospinning solution, and small-diameter and uniform nanofiber dressings were prepared by optimizing the PVP, SD, water, and ethanol in a ratio of 4.0:0.1:4.8:1.2. Subsequently, SC was mixed with the electrospun solution and encapsulated during electrospinning. The optimized composition of the electrospun bead-free nanofibers had an average diameter of 624.7 ± 99.6 nm. To characterize wound-dressing properties and performance from physicochemical and mechanical perspectives, SEM, DSC, XRD, FTIR, tensile strength, and elongation at break measurements were used to confirm that there was no interaction between SC, PVP, and SD. Additionally, a release test of the active substance showed that 100% of the SC was released within 30 min. Thus, PVP/SD-based composite nanofiber dressings containing SC may be useful for SC delivery. Overall, our study showed that PVP/SD composite nanofibers containing SC prepared using the electrospinning method are promising as skin wound dressings.

## Figures and Tables

**Table 1:** SC-loaded SD/PVP nanofiber composition.

Ingredients (g)	I	II	III	IV	V	VI	VII
SC	-	-	-	-	-	-	3.0
PVP	4.0	4.0	4.0	4.0	4.0	4.0	4.0
SD	-	0.1	0.1	0.1	0.1	0.1	0.1
Water (Solvent)	-	-	5.7	5.4	5.1	4.8	4.8
Ethanol (Solvent)	6.0	6.0	0.3	0.6	0.9	1.2	1.2

## Data Availability

Data are available on request due to restrictions, e.g., privacy or ethical considerations.
